# A recombinant RNA bacteriophage system to identify functionally important nucleotides in a self-cleaving ribozyme

**DOI:** 10.1186/1743-422X-11-116

**Published:** 2014-06-20

**Authors:** René CL Olsthoorn

**Affiliations:** 1 Department of Molecular Genetics, Leiden Institute of Chemistry, Leiden University, PO box 9502 RA, Leiden, The Netherlands

**Keywords:** Ribozyme, RNA phage, Evolution, MS2, Hammerhead, RNA catalysis

## Abstract

**Background:**

RNA bacteriophages like Qbeta and MS2 are well known for their high mutation rate, short infection cycle and strong selection against foreign inserts. The hammerhead ribozyme (HHRz) is a small self-cleaving RNA molecule whose active residues have previously been identified by mutational analysis of each individual base. Here the functionally important bases of HHRz were determined in a single screening experiment by inserting the HHRz into the genome of MS2.

**Findings:**

The minimal HHRz of *satellite Tobacco ringspot virus* was cloned into the genome of RNA bacteriophage MS2. Sequence analysis of the surviving phages revealed that the majority had acquired single base-substitutions that apparently inactivated the HHRz. The positions of these substitutions exactly matched that of the previously determined core residues of the HHRz.

**Conclusions:**

Natural selection against a ribozyme in the genome of MS2 can be used to quickly identify nucleotides required for self-cleavage.

## Background

The small RNA bacteriophages like Qbeta and MS2 are well known for their high mutation rate and short infection cycle [1]. These properties make RNA phages ideal candidates for studying evolution-related issues. Previously, an infectious cDNA clone of MS2 was constructed and used to monitor the response of MS2 to mutations in its genome [2]. As the wild-type MS2 cDNA clone produces ~10^12^ phages per ml culture after overnight growth in *E. coli.,* it was possible to reveal extremely rare evolutionary events like the repair of a 19-nt deletion [3]. Certain positions of the MS2 genome are quite tolerant to the insertion of foreign RNA of sizes up to 68 nt, however, when they contain target sites for cellular RNases, mutants rapidly emerge in which these sites have been destroyed [4,5]. So, I reasoned that the MS2 system could also be used to monitor the inactivation of a self-cleaving ribozyme. Since these ribozymes require a specific set of nucleotides for cleavage of the phosphodiester bond I expected that mutants would quickly arise to rescue the MS2 genome from cleavage. In contrast to conventional time-consuming mutation analysis, sequence analysis of the mutants would immediately show which nucleotides are important for cleavage. This is exactly what happened.

## Results

**Figure 1:**
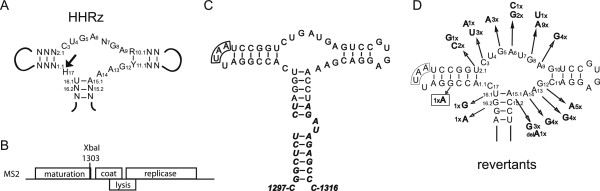
**Construction and evolution of a ribozyme in phage MS2. A**. Consensus sequence and structure of the minimal hammerhead ribozyme. N, any base; H = A, C, or U; Arrow indicates site of cleavage. **B**. Map of the MS2 genome. The XbaI-site was used for insertion of the ribozyme sequence. **C**. *satellite Tobacco ringspot ribozyme* (regular font) lacking the native loop-loop interaction inserted in MS2 RNA (bold italics). The UAA stop codon of the extended maturation gene is boxed. **D**. Overview of revertants and number of occurrences. The change marked by a rectangle occurred in combination with a C3- > U mutation.

**Figure 2:**
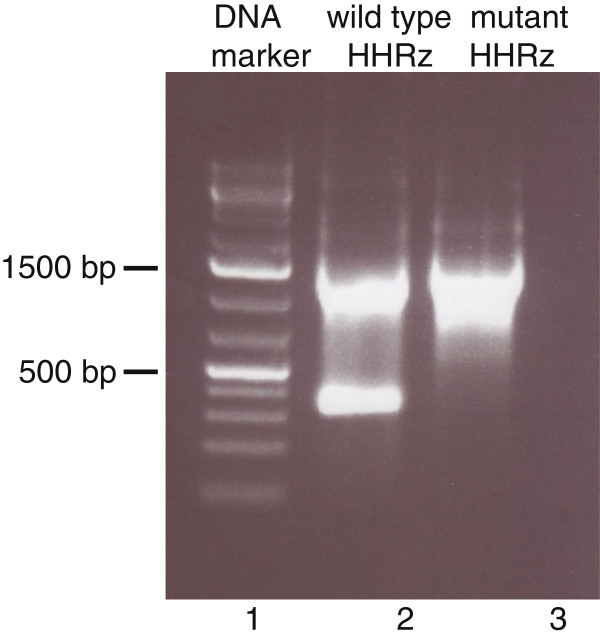
**Agarose gel showing self-cleavage of the HHRz in the context of MS2 RNA sequences.** Lane 1, DNA marker consisting of 75, 200, 300, 400, 500, 750, 1000, 1500, etc. base-pair fragments. Lane 2 and 3, products of T7 RNA polymerase directed transcription of templates containing the active and inactive (G5A mutant) HHRz, respectively.

The hammerhead ribozyme (HHRz), one of the best-studied ribozymes, was originally discovered in plant virus satellite RNA [6] and viroids [7], but appears to be present in all three kingdoms of life [8]. Its catalytic center consists of several conserved bases surrounded by three stem elements (Figure 1A). The role of the conserved residues has been solved by mutating each nucleotide individually and measuring its effect on cleavage [9]. Here, the minimal *satellite Tobacco ringspot virus* HHRz and an inactive control were cloned into MS2 cDNA (Figure 1B,C). Activity of the HHRz in the context of the MS2 genome was demonstrated by gelelectrophoretic analysis of in vitro synthesized transcripts. Figure 2 shows the self-cleavage of a 2334 nt MS2-luciferase hybrid RNA (see Methods) containing the active HHRz (lane 2). Expected products are 1886 and 448 nts. The inactive HHRz (lane 3) showed no signs of cleavage. Upon prolonged electrophoresis the size difference between the 1886 nts and 2334 nts RNAs of the active and inactive HHRz became better visible (not shown).

**Figure 3:**
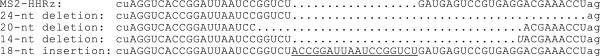
**Sequence of deletion and insertion mutants.** The sequence of the starting MS2-HHRz construct is shown on top. MS2 sequence is shown in lower case, HHRz sequence is shown in capitals. An 18-nt insertion, representing a partial duplication of the HHRz sequence, is underlined. A non-templated U residue in the 14-nt deletion variant is shown in italics.

Phages produced by the infectious clones were plated onto lawns of *E. coli* to yield separate plaques. Sequence analysis of individual plaques from the inactive HHRz containing phage revealed no sequence changes (13 out of 13 samples). Of 50 plaques taken from the active HHRz clone, 44 had acquired a single mutation, one had two mutations, and one had a single A-deletion (Figure 1D). The remaining four plaques had undergone large deletions of the HHRz or an insertion that presumably led to inactivation of the ribozyme (Figure 3).

All other changes (Figure 1) affected either the conserved core residues or directly adjacent base pairs (e.g. U16.1- > G or U2.1- > C). Mutation of C3, G5, G8, G12, A14, and A15.1 are known to reduce activity of the HHRz more than 100-fold, while mutation of A6, A9 and A13 result in 10–100 fold inhibition [9]. The absence of mutations at residues U4, U7, and C17, is in accordance with a previously observed C-variation at position 4 [10], the spacer function of position 7, and the observation that C17 can be anything but a G. The lack of mutations in the distal parts of the stems indicates that single mismatches have only minor effects on cleavage, as illustrated by the G1.4- > A change accompanying the C3- > U mutation in one clone.

It should be noted that although the C-terminus of the A protein was extended by three amino acids there appeared to be no strong selection pressure on this extension as none of the mutations affected the length of the A protein and only one mutation (U16.1G) led to an amino acid change in the extension (Ser to Ala). These data are in agreement with an earlier study on C-terminal extensions of the A protein [11].

## Discussion

The data show that natural selection against a ribozyme in the genome of MS2 can be used to quickly identify nucleotides that contribute to self-cleavage: the selected mutants give an accurate description of the previously determined core residues of the HHRz. Of course to obtain a complete overview of all four possible bases at each position, especially the less frequent base transversions, one needs to analyze substantially larger numbers of mutants.

The natural TRSV HHRz has a loop-loop interaction that increases catalytic activity over 100-fold [12-14]. Although the 100-fold higher cleavage rate would probably decrease the titer of the infectious clone to a similar extent, the remaining 10^6^ or 10^7^ plaque forming units per ml would suffice to screen for mutants that may have lost this loop-loop interaction.

The present method can be used to investigate other ribozymes, provided that their size is smaller than about 100 nts, they possess a large degree of secondary structure - MS2 displays a strong selection against large single-stranded inserts [11] – and cleave within the duration of one infection cycle, approximately 20 min. Combined with modern deep-sequencing methods it will allow the rapid examination of large numbers of mutants, widening the scope of this phage-based system.

## Methods

### Construction of mutants

pMS2000 contains a complete cDNA copy of the phage MS2 genome under transcriptional control of a thermoinducible promoter and produces 10^12^ plaque forming units (pfu) per mL culture after overnight growth [2]. The unique XbaI site at position 1303 of the MS2 genome was used to clone complementary sets of oligonucleotides that correspond to the sequence of the minimal STRV HHRz and an inactive variant containing the G5- > A mutation. The titers of the active HHRz construct and the inactive variant were about 2000 and 100 times, respectively, lower than that of the wild-type MS2 clone.

### Ribozyme activity assay

DNA fragments covering the region 964–1421 of the MS2 genome were produced by PCR on the pMS2000 variants containing the active and inactive variant of the HHRz using primers MS2up3 (GTATA*GCTAGC*GTAATACGACTCACTATAG**GGTATCTTGAACCCACTAGG**, MS2 sequence homologous to nts 964–983 in bold, NheI restriction site in italics) and MSLuc2 (TCCAGCTC*GGAT****CC*CGTTAGCGAAGTTGCTTGGGGCGACAG** (MS2 sequence complementary to nts 1393–1421 in bold, BamHI restriction site in italics). The two PCR fragments were digested with NheI and BamHI and cloned into a Firefly luciferase reporter vector (details will be described elsewhere). After linearization of the resulting plasmids with ApaI and purification of the DNA, RNA was synthesized by T7 RNA polymerase transcription using the RiboMAX Large Scale RNA Production System (Promega). Products were assayed by electrophoresis on an Ethidium bromide containing agarose gel.

### Phage growth and sequence analysis

Phages were plated onto lawns of *E. coli* strain XL-1 Blue MRF’ (Stratagene, Waldbronn, Germany) which contains a kanamycin selectable F-factor, needed for phage attachment. Individual plaques were lanced with a yellow tip and transferred to cell culture tubes containing 1 mL of LB supplied with kanamycin and F^+^ cells (OD650 ~ 0.05) and grown overnight at 37˚C. Phage present in 1 mL of supernatant were precipitated with PEG and phage RNA was extracted as described [2]. cDNA was synthesized using primer Dui59 (5’CCCCGTTAGCGAAG, complementary to nts 1409–1422 of the MS2 genome) and AMV Reverse Transcriptase (Promega, Benelux) and amplified by PCR using Taq polymerase (Fermentas, St. Leon-Rot, Germany) and primers Dui59 and Dui715. (5’CCCAAATCTCAGCCATGCATCGAGG, homologous to nts 1181–1205 of the MS2 genome). The DNA fragments were checked by agarose gel-electrophoresis and purified by ammonium acetate/isopropanol precipitation to remove unincorporated primers and deoxynucleotides. The fragments were sequenced using primer Dui59 and BigDye terminator chemistry (LGTC, Leiden, The Netherlands).

## Competing interests

The author declares that he has no competing interests.

## Authors’ contributions

RO did all the experiments and wrote the manuscript.
